# Individualism and the Extended-Self: Cross-Cultural Differences in the Valuation of Authentic Objects

**DOI:** 10.1371/journal.pone.0090787

**Published:** 2014-03-21

**Authors:** Nathalia L. Gjersoe, George E. Newman, Vladimir Chituc, Bruce Hood

**Affiliations:** 1 Faculty of Education, The Open University, Milton Keynes, Buckinghamshire, United Kingdom; 2 School of Management, Yale University, New Haven, Connecticut, United States of America; 3 School of Psychology, Yale University, New Haven, Connecticut, United States of America; 4 Bristol Cognitive Development Centre, Bristol University, Bristol, Gloucestershire, United Kingdom; University of Queensland, Australia

## Abstract

The current studies examine how valuation of authentic items varies as a function of culture. We find that U.S. respondents value authentic items associated with individual persons (a sweater or an artwork) more than Indian respondents, but that both cultures value authentic objects not associated with persons (a dinosaur bone or a moon rock) equally. These differences cannot be attributed to more general cultural differences in the value assigned to authenticity. Rather, the results support the hypothesis that individualistic cultures place a greater value on objects associated with unique persons and in so doing, offer the first evidence for how valuation of certain authentic items may vary cross-culturally.

## Introduction

Traders in celebrity memorabilia, such as Darren Julien—founder and president of one of the world's largest celebrity memorabilia auction houses - have noted that despite an increasing demand for investment opportunities in Eastern countries, interest in memorabilia is not yet as strong as it is in the United States and may be driven by very different motivations (personal communication). One explanation for this difference may be that Eastern collectors are simply less interested in U.S. celebrities. The current study, however, explores an alternative hypothesis that differences in the valuation of these items arise from more fundamental differences between individualist and collectivist cultures [1].

In what is commonly referred to as the “extended-self hypothesis,” James, Belk and Dittmar [2]–[4] suggest that people's self-concept goes beyond their physical body and cognitive processes to include all objects that they regard as “their own”—their friends and family, pets, hobbies and the objects that they own and use. As such, Belk (1988) explains how we become ‘contaminated’ in both positive and negative ways through contact with people's possessions (see also [4]–[7]). Recently, these notions of the extended-self and contamination (or, contagion) have been used to explain people's desire to own celebrity memorabilia in the West. Newman, Diesendruck and Bloom [8] demonstrate that consumers' valuation of celebrity memorabilia is explained by the belief that some immaterial quality or ‘essence’ of the celebrity has been transferred to the object via physical contact. For example, individuals who are more sensitive to contagion express a stronger desire to own positive celebrity memorabilia, and a stronger repulsion from negative celebrity memorabilia. This work builds upon a larger literature on “magical thinking,” which has demonstrated belief in contagion in both primitive cultures [9] and scientifically educated adults [6], [7], [10]–[12].

However, the generalizability of the extended-self concept across cultures remains unclear. Past research has demonstrated that the importance that people place on the “self” may be greatly influenced by cultural factors. Markus and Kitayama [13]–[15] propose two forms of self-concept: an independent view of self, prominent in individualist countries such as the U.S., and an interdependent view of self, prominent in collectivist cultures such as those found across South and East Asia. Typically, the independent view of self seeks autonomy from others and reveres unique individualism, while the interdependent view of self is concerned with social cohesion and avoids favoritism [1], [16]. This difference in emphasis on the individual raises the question, if celebrity memorabilia is valued because of its connection to a unique person, will it be valued less in cultures that place less emphasis on individuals?

The current study reports data collected from North American (U.S.) and South Asian (Indian) respondents. On the bipolar dimension of collectivism-individualism [1], the U.S. historically scores at the top of individualism scales (relative to all other countries), and India typically scores towards the collectivist end [17]–[22]. This relative difference persists regardless of the socio-economic status or level of education of the respondents [23].

The primary goal of this paper was to explore how valuation of authentic items may vary as a function of differences between individualist and collectivist cultures. Respondents were told that an item was placed in a specialized machine that made an identical copy of it (no explanation was provided as to the mechanism). Respondents then estimated the value of the original and the duplicate. Between-subjects we varied the type of object: Half of the participants valued “contagion objects,” such as artworks or celebrity memorabilia, which previous research has tied to a belief in contagion [8], [24]. The other half evaluated authentic items that are valued for reasons unconnected to individual persons. We hypothesized that if members of individualist cultures value “extensions of self” [3] more than members of collectivist cultures, U.S. respondents should value “contagion” originals more than Indian respondents, while both cultures should value other types of authentic objects roughly the same.

## Methods

### Participants

Two-hundred-and-forty-one adults (41% female, 118 U.S, 124 Indian) were recruited using Amazon's online Mechanical Turk (mTurk) service. mTurk is a database of over 100,000 users, who complete short tasks for monetary compensation [25]. Respondents were randomly assigned to one of eight conditions in a 4 (Object)×2 (U.S. vs. India) between-subjects design.

### Ethics Statement

In this and the subsequent internal replication, written informed consent was obtained before the respondents commenced and full ethical approval for the studies was received from the Faculty of Arts and Sciences Human Subjects Committee at Yale University. Respondents were classified as being from India or the USA if the questionnaire source and stated ethnicity were the same.

### Materials

Each respondent was asked to imagine that physicists had created a duplicating machine that could make exact copies of any object placed inside. Philosophers have long been intrigued by perfect duplicates [26]–[28] and thought scenarios like this have been widely used to examine intuitions about authenticity and identity, primarily in North American populations. Participants were told that an object with a specific market value was placed inside Pod A, the machine was activated, and then respondents were shown an illustration of identical objects in Pod A and Pod B [29], [30]. Half of the respondents were presented with one of two “contagion” objects (a painting by a famous artist or a sweater owned by President John F. Kennedy (JFK)). The other half of the participants were presented with one of two “distance” objects [31] - objects valued because they originated distantly in time (Dinosaur Bone) or in space (Moon Rock). Each respondent viewed only one object. All respondents were then asked to estimate the monetary value of both the original and the duplicate. Responses were made using a slider bar with $0 and the value of the original (Painting = $1M; Moon Rock, Dinosaur Bone and Sweater = $10,000) as end-points.These values were based on the approximate market value for the “authentic” version of each item type.To normalize scores across items, all values were subsequently converted to percentages. Additionally, respondents reported the personal value (*How much would you like to own this item?*), and the perceived social value (*Does this item belong in a museum?*) of each item on a scale from 0 = not at all to 100 = very much so. The three measures of value (monetary, personal, social) were highly correlated (α = .77) and were averaged to produce one measure of value each for the original and the duplicate.

## Results and Discussion

**Figure 1:**
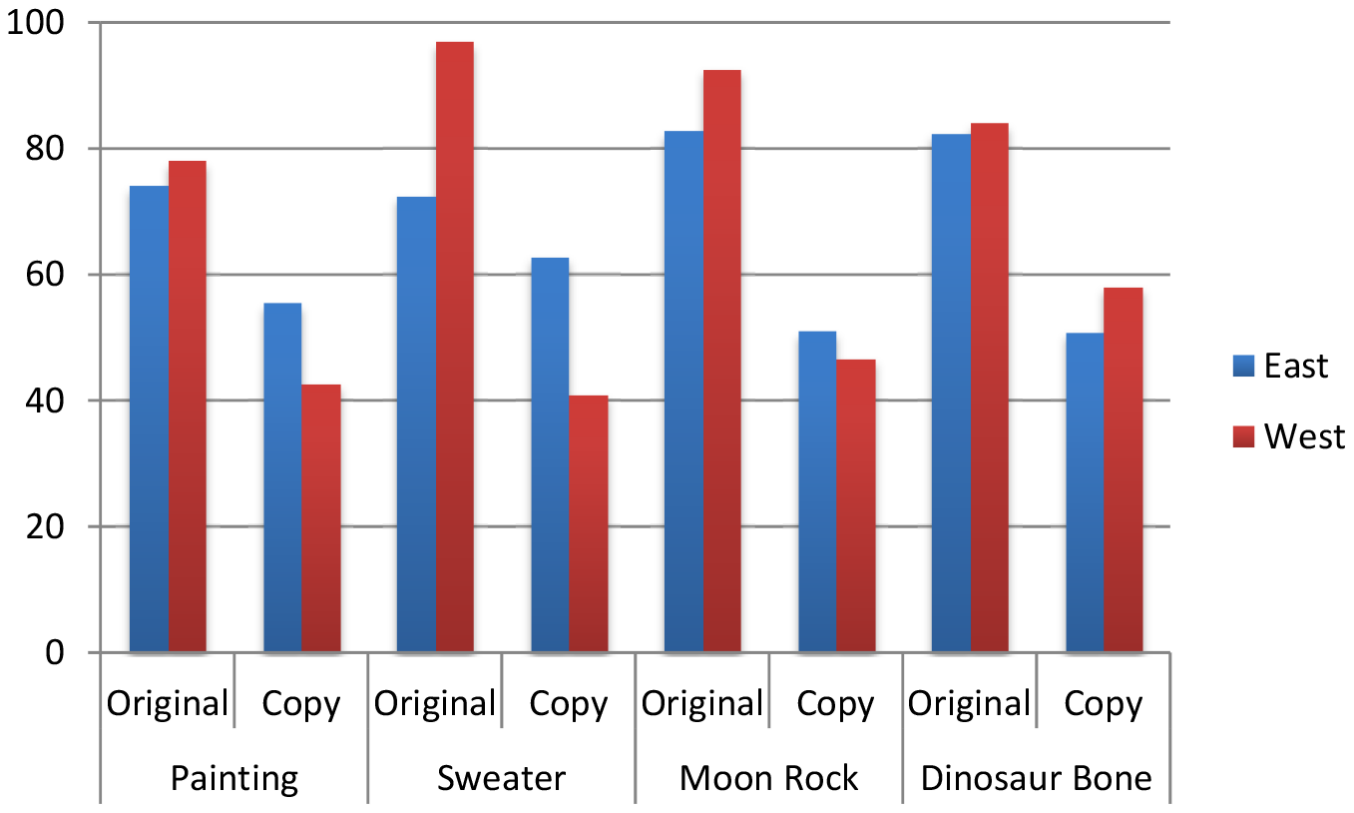
Mean percentage valuation of original and copy of each object (Painting, Sweater, Moon Rock and Dinosaur Bone) by respondents from India (East) and America (West).

Planned t-tests revealed no significant effect of participant gender so data were collapsed along this variable. A mixed-model ANOVA with 2 (Culture: U.S. vs. India)×4 (Object: Sweater, Artwork, Dinosaur Bone, Moon Rock) as between-subject variables and 2 (Authenticity: Original vs. Copy) as a within-subject variable indicated a significant three-way interaction, *F*(3, 234) = 4.44, *p*<.01. We conducted a series of repeated-measures ANOVA to determine the nature of this interaction for each object (Figure 1).

### Painting

We observed a significant interaction between Culture and Authenticity, *F*(1, 58) = 8.74, *p*<.01. U.S. and Indian respondents both valued the original Painting (U.S.: *M* = 75.81, *SD* = 28.46, Indian: *M* = 69.86, *SD* = 23.16) significantly more than the identical duplicate (U.S.: *M* = 38.53, *SD* = 29.73, Indian: *M* = 56.96, *SD* = 21.17), U.S.: *t*(28) = 4.96, *p*<.001, *r* = .7, Indian: *t*(30) = 3.49, *p*<.01, *r* = .5 but this difference was significantly lower for Indian respondents, as indicated by the significant interaction, *t*(236) = 3.63, *p*<.001, *r* = .2.

### Celebrity Sweater

We observed a significant interaction between Culture and Authenticity, *F*(1, 58) = 22.16, *p*<.001. U.S. respondents valued the original Sweater (*M* = 78.50, *SD* = 18.79) significantly more than the identical duplicate (*M* = 28.59, *SD* = 31.41), *t*(29) = 7.28, *p*<.001, *r* = .8 but Indian respondents did not (Original: *M* = 59.89, *SD* = 23.47, Duplicate: *M* = 49.06, *SD* = 19.01), *t*(29) = 1.61, *p* = .12.

### Distance-objects

In contrast to the two “contagion” objects, we only observed a main effect of Authenticity for the Dinosaur Bone, *F*(1, 58) = 82.31, *p*<.001 and Moon Rock, *F*(1, 60) = 51.39, *p*<.001, and no interaction with culture, Dinosaur Bone- *F*(1, 58) = 1.97, p = 0.17; Moon Rock- F(1, 60) = 0.39, p = 0.54). Both U.S. and Indian respondents valued the original Dinosaur Bone (U.S.: *M* = 83.56, *SD* = 14.00, Indian: *M* = 72.97, *SD* = 22.59) and Moon Rock (U.S.: *M* = 79.77, *SD* = 21.67, Indian: *M* = 75.99, *SD* = 20.79) significantly more than the identical duplicates (Dinosaur Bone – U.S.: *M* = 43.94, *SD* = 27.97, Indian: *M* = 45.00, *SD* = 21.80; Moon Rock– U.S.: *M* = 48.97, *SD* = 20.79, Indian: *M* = 43.97, *SD* = 26.62), Dinosaur Bone - U.S.: *t*(28) = 6.80, *p*<.001, *r* = .8 Indian: *t*(58) = 4.32.56, *p*<.001, *r* = .6, Moon Rock- U.S.: *t*(29) = 3.93, *p*<.001, *r* = .6, Indian: *t*(30) = 4.89, *p*<.001, *r* = .7. Cross-cultural valuation differences were not significant *t*(60) = −0.14, *p* = 0.89.

In sum, U.S. respondents valued authenticity for all of the different objects — in all cases they rated the original object as substantially more valuable than the duplicate. In contrast, Indian respondents valued the authenticity of “distance objects” in a similar manner to U.S. respondents, but were less concerned with the authenticity of “contagion” objects. These results are consistent with the notion that cross-cultural differences in valuation are rooted in the value placed on extensions of the self (contagion objects), rather than the value associated with authenticity more generally.

### Internal Replication

We conducted a follow-up experiment with a new sample of 609 adult respondents (39% female, 315 U.S., 294 Indian). This experiment was very similar to Experiment 1 except that respondents were told that the sweater belonged to their favorite living celebrity (rather than JFK) and we equated the value of all the original items ($10,000). The first change was to control for the possibility that Indian respondents did not know who JFK was or that they simply didn't value items that had belonged to an American celebrity. By asking them to think of the original sweater as belonging to their favorite *living* celebrity we hoped to circumvent this possible confound while controlling for the financial effect that the death of a celebrity has on the value of their belongings. The second methodological change was to ensure that the results of Experiment 1 were not driven by differences in the absolute value of the original. For simplicity, this study only measured estimations of monetary value.

In short, the results replicated those obtained in the previous experiment. A mixed-model ANOVA revealed a significant three-way interaction between Culture, Authenticity, and Object, *F*(3, 601) = 3.64, *p*<.05. As in the previous experiment, there was a significant two-way interaction between Culture and Authenticity for valuation of the Painting, *F*(1, 156) = 7.67, *p*<.01, and valuation of the Sweater, *F*(1, 148) = 10.40, *p*<.01. In contrast, for the two “distance” objects, there was only a main effect of Authenticity, Dinosaur Bone: *F*(1, 156) = 52.81, *p*<.001, Moon Rock: *F*(1, 141) = 36.38, *p*<.001, and no interaction with Culture, Dinosaur Bone – *F*(1, 156) = 0.47, *p* = 0.49; Moon Rock – *F*(1, 141) = 1.12, *p* = 0.3. The one difference between the original study and the replication was that Indian respondents valued the original celebrity Sweater higher than the copy, *t*(71) = 5.19, *p*<.001, *r* = .5. However, the difference in valuation between the original and copy of the contagion items was again significantly higher for U.S. than Indian respondents, Painting: *t*(156) = 2.77, *p*<.01, *r* = .2, Sweater: *t*(147) = 3.25, *p*<.01, *r* = .3. Thus, removing confounds associated with JFK and the different item values had no effect on the overall pattern of results. Both U.S. and Indian respondents valued authentic originals more than copies across items, but U.S. respondents valued the original contagion items significantly more than Indian respondents.

## General Discussion

The primary goal of this paper was to explore how valuation of authentic items may vary as a function of culture. In the experiment and its replication, we found that U.S. respondents placed significantly more value than Indian respondents on authentic contagion items associated with individual persons. By comparison, no cultural differences were found for objects valued because of their distant origins (in time or space). This is consistent with the hypothesis that collectivist cultures are less focused on the individual and therefore place less monetary worth on authentic objects that are valued for their connections to particular individuals.

One concern might be that the currency of valuation (dollars) was kept the same across respondent groups. We did this because Frazier et al. [31] found that using local currencies left open the possibility that participants used the same anchor points (e.g. 1 million dollars or pounds) regardless of the difference of those amounts relative to each other, making comparison more difficult. Dollars are widely used in India so we expected that respondents would have a clear idea of what they were worth. Nevertheless, this raises the alternative possibility that cultural differences in valuation arose because a dollar in India will buy a great deal more than a dollar in the U.S. [32]. We argue that this alternative cannot explain the current results because 1) this should have been evident across the different object types (including distance objects), rather than just for the contagion items, and 2) in addition to the monetary value, we also asked about personal value (*How much would you like to own the item*) and social value (*Does this item belong in a museum?*) and these scores were correlated highly with the monetary valuation. If the difference in monetary valuation had been driven by a different concept of what a dollar was worth then we wouldn't expect non-monetary assessments of value to pattern in a similar manner.

An additional concern is that Indian respondents could have understood the paradigm differently from American respondents. For instance, they may have had a different concept of what was meant by a “perfect duplicate” – for instance, Indian respondents may have thought the copy was generally less valuable than American respondents. However, the fact that there was no significant difference in respondents' valuation of the distance objects (the Dinosaur Bone and Moon Rock) suggests that both groups understood the paradigm in the same manner but valued the contagion objects differently.

We interpret the cultural difference in valuation of celebrity items as evidence that respondents from India attribute less value to items associated with particular individuals. A somewhat different explanation is that Indian respondents are, overall, simply less sensitive to the concept of contagion. There is some empirical evidence to reject this account – for instance, Hejmadi, Rozin, & Siegal [33] point out that contamination and purity constitute major themes in Hindu culture and showed that Hindu Indian children aged 4–5 years are more sensitive to physical and spiritual contagion than equivalent American children, that they believe contamination is more indelible with regards to a range of sterilization procedures and that these biases become stronger with age. Meyer, Leslie, Gelman & Stillwell [34] conducted a study examining American and Indian adults' beliefs with regards to contamination through surgical transplants. Meyer et al did not specifically target Hindu Indians and, like us, recruited participants using MTurk. Nonetheless, they found that Indian adults extend contamination concerns further than American adults. Measuring concerns about transplants from morally positive and negative donors, Indian respondents expressed greater concern about a range of items including a skin graft and a pacemaker while American concerns focused just on transplants of internal, biological items such as a heart. These findings suggest that contamination beliefs are, if anything, stronger in Indian populations than in American ones and therefore fail to explain the pattern of results in the current study.

Authentic items, such as celebrity memorabilia, do not usually differ aesthetically or functionally from inauthentic duplicates and yet (in the West) we often place greater value on them because of their historical connection to a time, place or person [35], [36]. Rather than reflecting a lack of scientific education, there is increasing awareness that magical beliefs, such as valuing contagion, are an important part of everyday thought [8], [31], [37], [38] and arise early in the development of Western children [29], [33], [39].

The present experiments make two important contributions to this literature: Previous work has revealed that valuation of celebrity objects in the West is strongly motivated by contagion biases [3], [8], [24], [40]. To our knowledge, however, there has not been any previous attempt to examine whether this is culturally-specific. This paper provides the first empirical demonstration that contagion items are attributed greater monetary valuation in the West than in the East. Second, these results demonstrate that this difference is not driven by cultural differences in the value associated with originals (or authenticity) per se as both groups valued authentic items that were not connected to individual persons the same. Rather, the pattern of results suggests that items connected to individual persons are valued differently across the two cultures because individualism is valued more in the West than in the East and, by extension, so too are the objects that individuals own. These findings have important implications for our understanding of cultural variance in the extended-self concept and, more broadly, the psychological mechanisms underlying our preferences for authentic items.
